# Numerical Simulation in Microvessels for the Design of Drug Carriers with the Immersed Boundary-Lattice Boltzmann Method

**DOI:** 10.3390/mi16040389

**Published:** 2025-03-28

**Authors:** Yulin Hou, Mengdan Hu, Dongke Sun, Yueming Sun

**Affiliations:** 1 School of Chemistry and Chemical Engineering, Southeast University, Nanjing 211189, China; 2 Key Laboratory of Structure and Thermal Protection of High Speed Aircraft, Ministry of Education, Southeast University, Nanjing 211189, China; 3 Jiangsu Key Laboratory for Design and Manufacture of Micro-Nano Biomedical Instruments, School of Mechanical Engineering, Southeast University, Nanjing 211189, China

**Keywords:** lattice Boltzmann method, immersed boundary method, blood dynamics, drug carrier

## Abstract

This study employs numerical techniques to investigate the motion characteristics of red blood cells (RBCs) and drug carriers (DCs) within microvessels. A coupled model of the lattice Boltzmann method (LBM) and immersed boundary method (IBM) is proposed to investigate the migration of particles in blood flow. The lattice Bhatnagar–Gross–Krook (LBGK) model is utilized to simulate the flow dynamics of blood. While the IBM is employed to simulate the motion of particles, using a membrane model based on the finite element method. The present model was validated and demonstrated good agreements with previous theoretical and numerical results. Our study mainly examines the impact of the Reynolds number, DC size, and stiffness. Results suggest that these factors would influence particles’ equilibrium regions, motion stability and interactions between RBCs and DCs. Within a certain range, under a higher Reynolds number, the motion of DCs remains stable and DCs can swiftly attain their equilibrium states. DCs with smaller sizes and softer stiffness demonstrate a relatively stable motion state and their interactions with RBCs are weakened. The findings would offer novel perspectives on drug transport mechanisms and the impact of drug release, providing valuable guidance for the design of DCs.

## 1. Introduction

In recent years, there has been a lot of interest in biomedical engineering regarding the development of drug delivery systems for targeted therapy. To address issues like drug instability and uncontrolled release, scientists have created many tiny drug carriers known as micro–nano drug carriers (DCs) [1,2,3,4]. It is essential to understand how these DCs move in blood vessels. It could contribute to improving drug delivery methods and enhancing treatment outcomes. DCs are usually carried through the bloodstream to different parts of the body. They offer advantages such as better drug solubility and stability, targeted delivery, controlled release, and the potential for combined therapies. On the other hand, red blood cells (RBCs) are the most abundant cellular components in our blood, known for their flexibility and long lifespan. When drug carriers enter the bloodstream, they interact with RBCs through collisions or adhesion [5,6]. Interestingly, some researchers suggest using RBCs themselves as carriers due to their unique characteristics [7,8]. The interaction between RBCs and DCs depends on various factors. Therefore, it is of great significance to study the migration and distribution of DCs with RBCs in blood vessels.

Nowadays, a variety of methodologies have been employed to investigate the migration of RBCs and the complex interactions between RBCs and DCs. Their strengths and limitations are listed in Table 1. Experimental techniques of in vivo and in vitro facilitate the detailed analysis of drug uptake, release kinetics, and intracellular drug distribution within RBCs. However, owing to the intricate nature of human systems, they are unfeasible to thoroughly investigate the physiological environment. Accordingly, computational fluid dynamics (CFD) has emerged as a reliable tool for examining the behavior of DCs within microvessels [9,10,11,12]. Currently, researchers have also proposed combining computational simulation with artificial intelligence to assist in the efficient and precise selection and design of drug carriers [13,14,15].

In CFD, discrete methods are extensively employed in the micro–environment, involving the dissipative particle dynamics (DPD) [21,22], smoothed particle hydrodynamics (SPH) [23,24], and lattice Boltzmann method (LBM). DPD is conducive to simulating macroscopic systems and soft matter systems. However, it necessitates the configuration of a multitude of parameters pertaining to particle interactions, thereby heightening the complexity of simulations [25]. SPH is apt for simulating multiphase fluid dynamics and excels at addressing collision scenarios. However, it faces challenges in handling boundary conditions and exhibits a relatively reduced computational efficiency [26]. In contrast, LBM exhibits great potential in the simulation of complex fluid–solid problems due to its excellent computational efficiency and ability. As a result, the simulation method adopted in this study is LBM, integrated with the immersed boundary method (IBM). IBM was initially introduced by Peskin [27] in 1972 to simulate the intricate dynamics of blood flow within the human heart. Researchers have carried out a series of work on the simulations of RBCs in blood vessels using LBM. More representative studies are summarized below. Sun et al. [28] introduced the presence of white blood cells (WBCs) in the bloodstream through this method, and subsequently calculated the augmentation in resistance attributable to RBC rolling and adhesion. Their team further refined the model to explicitly incorporate both cell–cell and cell–wall interactions [29]. Afterwards, Skorczewski et al. [30] utilized a two–dimensional IB–LBM to investigate the movement of platelets in proximity to a vessel wall and near an intravascular thrombus. This research showed that the tumbling of platelets in the RBC–depleted zone was strongly influenced by nearby RBCs. A two–dimensional simulation using IB–LBM was performed by Kaoui [31] to study drug release from liposomes into the bloodstream. The research considered the impact of surrounding RBCs on drug release, especially at large Schmidt numbers. Ye et al. constructed a model within LAMMPS, incorporating LBM, IBM, and spring network (SN), to simulate the dynamics of a broad spectrum of particles in blood flow. Firstly, they examine the effect of cell stiffness on adhesion dynamics under shear flow [32]. Then, they used this model to simulate the migration of magnetic particles within blood flow [33]. They further simulated the migration of RBCs and particles depending on the shape of particles, shear rate of blood flow, and the narrowing degree of microvessels [34,35,36]. Yue et al. [37] developed a hybrid immersed boundary and a coupled double–distribution–function lattice Boltzmann method to simulate the transport and adhesion of thermogenic nanocarriers in microvessels. Djukic et al. [26] integrated IB–LBM to simulate the movement of both rigid and deformable particles within arteries characterized by stenosis and bifurcation. Nikfar et al. [38] used the method coupling of LBM, SN, and frictional immersed boundary method to simulate the desorption of particles from the surface of RBCs in microvessels under shear flow.

For the current work, we intend to analyze the migration and distribution of individual DCs in a curved blood vessel. IB–LBM has the advantage of describing the interactions between RBCs and DCs easily and accurately. It also offers more flexibility in handling complex boundary conditions since most current simulations concerning DCs in microvessels are based on statistical methods to study the collective distribution behavior of these particles. The problem description and numerical methodology of our work are stated in Section 2. IB–LBM has been effectively employed due to its superior capabilities in calculating flow fields and fluid–structure coupling. Section 3 presents the discussions and results of the simulations conducted under varying conditions, involving the effects of the Reynolds number of the vessel, and the size and stiffness of DCs. Finally, Section 4 concludes the paper with a summary of the findings and outlines the directions for future research. Considering that nanometric DCs have been extensively investigated through simulation, our study primarily concentrates on the examination of micrometric DCs.

## 2. Numerical Methods

### 2.1. Problem Statement

**Figure 1:**
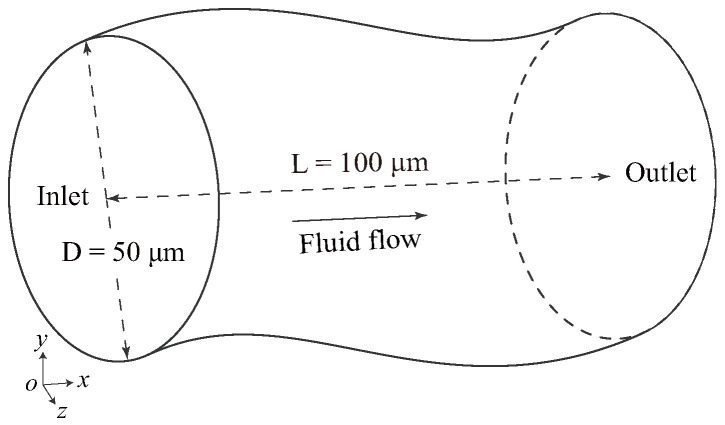
Illustration of the curved vessel with a length of 100 μm in the *x* direction and a diameter of 50 μm.

The objective of the simulation is to investigate the migration and distribution of DCs within the vessel. Given that most blood vessels in the human body are not linear, we opted for a three–dimensional curved channel as our research model. And the schematic diagram is shown in Figure 1. In the vessel model, the length in the *x* direction *L* is 100 μm and the inner diameter *D* is 50 μm. It maintains the same diameter even at the bends of the model.

In large vessels such as the aorta, blood flow is Newtonian flow. This allows the blood to maintain a relatively stable flow under normal physiological conditions. Conversely, blood viscosity varies with the shear rate, displaying non–Newtonian behavior in capillaries. Non–Newtonian blood conforms to the power–law model. The simulated vascular scale in this study is microarterioles and microvenules, representing an intermediate regime between the aforementioned scenarios. In the simulation, in order to simplify the model, blood is considered as a Newtonian fluid, which is characterized by the density ρ of 1050 kg/m^3^ and dynamic viscosity μ of $1.2\times 10^{-3}$ Pa·s [35]. Blood flow is driven by the pressure difference Δp between the inlet and outlet of the vessel. This can be calculated through equation $\Delta p=\rho a_{x}L+p_{in}-p_{out}$, where $a_{x}$ is the acceleration of fluid in the *x* direction. The periodic boundary condition in *x* direction is also implemented to simulate the flow within an infinite channel. This implies that particles exciting the vessel will re-enter from the inlet. The particles retain their properties and can be expressed as $P_{p}\left(0,y,z,t+\Delta t\right)=P_{p}^{\prime }\left(L,y,z,t\right)$, where $P_{p}$ represents the properties of particles. The Reynolds number in the system is defined by the formula $Re=\rho U_{m}D/\mu$, where $U_{m}$ is the mean velocity of the flow field. The expression for the mean velocity is $U_{m}=\Delta pD^{2}/32\mu L$. It should be emphasized that $U_{m}$ serves as an approximate value to estimate the flow field, rather than representing the real velocity. At the initial stage, RBCs and DCs are randomly released within the vessel. The fluid both inside and outside the particles is characterized by consistent density and viscosity.

### 2.2. Lattice Boltzmann Method for Blood Flow

Since the blood is considered as an incompressible fluid, the macroscopic governing equation for the flow field, with an external force term F can be calculated as follows:

(1) $$\begin{array}{c} \frac{\partial \rho }{\partial t}+\nabla \cdot \left(\rho u\right)=0, \end{array}$$

(2) $$\begin{array}{c} \rho \frac{\partial u}{\partial t}+\rho \left(u\cdot \nabla \right)u=-\nabla p+\mu \nabla^{2}u+F, \end{array}$$

where ρ, u, *p*, μ are the density, velocity, pressure, and dynamic viscosity of the blood flow, respectively.

In this work, we utilize the lattice Boltzmann method (LBM) to address the Navier–Stokes equation. We employ the standard single–relaxation–time lattice Bhatnagar–Gross–Krook (LBGK) model to solve the LB equation. The governing equation, inclusive of the external force term, is presented as follows:

(3) $$f_{i}\left(x+e_{i}\delta t,t+\delta t\right)-f_{i}\left(x,t\right)=-\frac{1}{\tau }\left[f_{i}\left(x,t\right)-f_{i}^{eq}\left(x,t\right)\right]+\delta tF_{i},$$

Here, $f_{i}\left(x,t\right)$ is the density distribution function, representing the probability of finding a pseudo fluid particle moving in the *i*-th direction on the discrete lattice at position x and time *t*, $f_{i}^{eq}\left(x,t\right)$ is the equilibrium distribution function, $e_{i}$ is the discrete velocity of the pseudo fluid particle, δt is the time step, and τ is the relaxation time. $f_{i}^{eq}$ represents the discretized equilibrium distribution function derived from the Maxwell–Boltzmann equilibrium and is defined as follows:

(4) $$f_{i}^{eq}=\rho \omega_{i}\left[1+\frac{e_{i}\cdot u}{c_{s}^{2}}+\frac{{\left(e_{i}\cdot u\right)}^{2}}{2c_{s}^{4}}-\frac{u^{2}}{2c_{s}^{2}}\right],$$

In this work, we employ the three–dimensional 19–velocity (D3Q19) model. The 19 discrete velocities of this model, denoted as $e_{i}$, are given by

(5) $$\left.\left[e_{i},i=0,\ldots ,18\right]=c\left[\begin{array}{c} 0,1,-1,0,0,0,0,1,-1,-1,1,-1,1,1,-1,0,0,0,0\\ 0,0,0,1,-1,0,0,0,0,-1,1,0,0,-1,1,-1,1,-1,1\\ 0,0,0,0,0,1,-1,1,-1,0,0,1,-1,0,0,1,-1,-1,1 \end{array}\right.\right],$$

where *c* is the lattice speed. It can be expressed by c=Δx/Δt. The force term $F_{i}$ in Equation (3) can be expressed by

(6) $$F_{i}=\left(1-\frac{1}{2\tau }\right)\omega_{i}\left(\frac{e_{i}-u}{c_{s}^{2}}+\frac{e_{i}\cdot u}{c_{s}^{4}}e_{i}\right)\cdot F,$$

where weight coefficients $\omega_{0}=1/3$, $\omega_{1-6}=1/18$, $\omega_{7-18}=1/36$ and the sound speed term $c_{s}=c/\sqrt{3}=\Delta x/\sqrt{3}\Delta t$. As the density distribution $f_{i}$ for each time step is known, the physical quantities of fluid density ρ and velocity u can be derived using the following equations:

(7) $$\begin{array}{c} \rho =\sum_{i}f_{i}, \end{array}$$

(8) $$\begin{array}{c} \rho u=\sum_{i}e_{i}f_{i}+\frac{1}{2}F\Delta t, \end{array}$$

The kinematic viscosity of the fluid ν is related to the relaxation time τ and can be expressed by

(9) $$\nu =\left(\tau -\frac{1}{2}\right)c_{s}^{2}\Delta t,$$

In the LBM, setting boundary conditions is crucial, as they can significantly influence both numerical stability and computational accuracy. In this study, the periodic boundary condition and pressure boundary condition are applied to the inlet and outlet of the blood vessel model, which are discussed in Section 2.1. A no–slip boundary condition is also imposed on the vessel wall to ensure that the velocity of the particle boundary aligns with that of the surrounding flow nodes.

### 2.3. Model of Red Blood Cells and Drug Carriers

**Figure 2:**
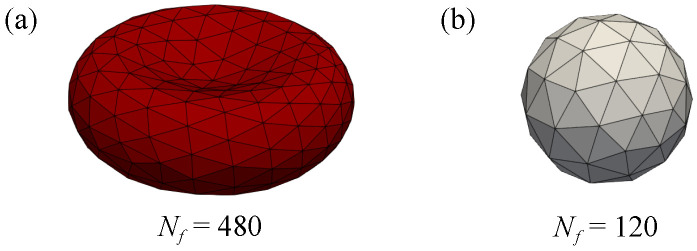
Illustrations of particle models which are divided into several triangular face elements: (**a**) RBC; (**b**) DC.

DCs can be divided in the forms of microspheres, nanospheres, nanotubes, dendrimers, liposomes, polymersomes, foams, hydrogels [39,40,41], and so on. These can be designed with tailored properties, including size, composition, surface chemistry, release kinetics [42]. They are also widely used in the pharmaceutical and medical fields [43]. In order to analyze the forces acting on the moving RBCs and DCs, we introduce a three-dimensional finite element membrane model utilizing the energy method [44]. In this model, both the RBC membrane and DC surface are divided into $N_{f}$ triangular face elements. These elements are generated by a number of *N* discrete Lagrange nodes. In addition, the quantity of triangular face elements and discrete vertices maintains a relationship defined by the equation $2N=N_{f}+2$. The resolution of the particles $N_{f}$ can be adjusted according to the spatial step and the accuracy required for the simulation. It is assumed that each element tends to maintain its flatness even under stress deformation. In the simulation, drug carriers are treated as spherical particles, while RBCs naturally exhibit a biconcave disk shape and possess a degree of deformability. In order to describe the initial model of the RBC, we project the mesh from the spherical surface using the subsequent equations [45]:

(10) $$x_{RBC}=Rx,y_{RBC}=\frac{1}{2}R{\left(1-r^{2}\right)}^{1/2}\left(C_{0}+C_{1}r^{2}+C_{2}r^{4}\right),z_{RBC}=Rz,$$

where $r^{2}=x^{2}+z^{2}$ and *R* is adjusted to preserve the volume. In this work, we adopt $C_{0}=0.207$, $C_{1}=2.003$, $C_{2}=-1.123$. Schematic diagrams of RBCs and DCs are shown in Figure 2. The radius of RBC and DC adapted in the model is 3.5 μm and 1.3–2.0 μm, respectively.

The potential energy of the particle, involving the in–plane strain energy $E_{S}$, the bending energy $E_{B}$, the surface energy $E_{A}$ and the volume energy $E_{V}$, is defined as follows

(11) $$E\left(x_{n}\right)=E_{S}+E_{B}+E_{A}+E_{V},\;n=0,1,2,...,N-1,$$

In this model, we apply the elastic law introduced by Skalak [46] to describe in–plane stretching and expansion. Face elements tend to be conserved all the time. The in–plane strain energy $E_{S}$ is the sum of in–plane strain energy density $\epsilon_{s,i}$ of each triangular element.

(12) $$\begin{array}{c} E_{S}=\sum_{i}^{N_{f}}\epsilon_{s,i}A_{i}, \end{array}$$

(13) $$\begin{array}{c} \epsilon_{s}=\frac{\kappa_{s}}{12}\left(I_{1}^{2}+2I_{1}-2I_{2}\right)+\frac{\kappa_{\alpha }}{12}I_{2}^{2}, \end{array}$$

where $A_{i}$ is the area of the *i*–th triangular element. The in-plane elastic shear modulus $\kappa_{s}$ and area dilation modulus $\kappa_{\alpha }$ are the control quantities of response to deformation. In Equation (13), $I_{1}=\lambda_{1}^{2}+\lambda_{2}^{2}-2$ and $I_{2}=\lambda_{1}^{2}\lambda_{2}^{2}-1$ are strain invariants, $\lambda_{1}$ and $\lambda_{2}$ are the local principal in-plane stretch ratios. The bending energy $E_{B}$ refers to the curvature contribution in the model, and can be calculated by

(14) $$E_{B}=\frac{1}{2}\kappa_{B}\sum_{i,j}^{N_{f}}\tan^{2}\left(\frac{\theta_{ij}-\theta_{ij}^{0}}{2}\right),$$

where $\kappa_{B}$ is the bending coefficient, and $\theta_{ij}$ represents the angle between the normal vectors of neighboring triangular elements. The sum runs over all pairs of adjacent face elements. Since the particle tends to conserve surface and volume throughout, we introduce the surface energy $E_{A}$ and volume energy $E_{V}$ into the model. The expressions are as follows:

(15) $$\begin{array}{c} E_{A}=\frac{1}{2}\kappa_{A}{\left(\frac{A-A_{0}}{A_{0}}\right)}^{2}, \end{array}$$

(16) $$\begin{array}{c} E_{V}=\frac{1}{2}\kappa_{V}{\left(\frac{V-V_{0}}{V_{0}}\right)}^{2}, \end{array}$$

where $\kappa_{A}$, $\kappa_{V}$, *A*, *V* are the area compressibility modulus, volume compressibility modulus, total surface area, and the total volume of the membrane, respectively. In addition, the symbol 0 indicates the equilibrium state. As the total energy $E(x_{n})$ is known, the fluid force acting on the Lagrange membrane node *n* at position $x_{n}$ can be computed by

(17) $$F\left(x_{n}\right)=-\frac{\partial E(x_{n})}{\partial x_{n}},\;n=0,1,2,...,N-1,$$

### 2.4. Immersed Boundary Method for Fluid–Solid Coupling

**Figure 3:**
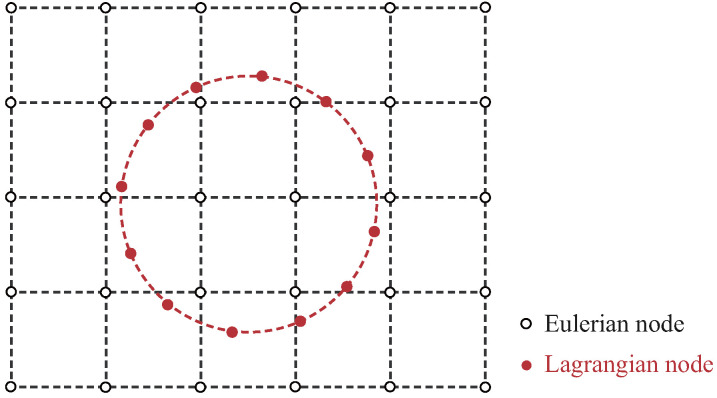
Illustration of Eulerian and Lagrangian meshes.

In this work, IBM is introduced to deal with the fluid–solid interfaces. It facilitates the simulation of fluid–structure interactions by characterizing drug carriers as Lagrangian particles within the fluid flow, which is dictated by LBM. This approach allows for a detailed examination of the complex interactions among drug carriers, blood circulation and vascular walls. As shown in Figure 3, Eulerian and Lagrangian meshes are used to denote position vectors of the fluid nodes $x_{f}$ and the moving immersed boundary nodes $x_{n}$, respectively. We employ the Dirac Delta function to convey information between the two meshes.

In the IBM, the particle–fluid interacting force $F(x_{n})$ is distributed to the adjacent fluid nodes $x_{f}$ by

(18) $$F\left(x_{f}\right)=\sum_{n}F\left(x_{n}\right)\delta \left(x_{n}-x_{f}\right),$$

where δ(·) is the Dirac delta function. In this work, two-point interpolation is used to transfer data between these two meshes.

(19) $$\begin{array}{c} r=x_{f}-x_{n}, \end{array}$$

(20) $$\begin{array}{c} \delta \left(r\right)=\phi \left(x\right)\phi \left(y\right)\phi \left(z\right),\;\phi \left(r\right)=\left\{\begin{array}{cc} 1-|r| & |r|<1\\ 0 & |r|\geq 1 \end{array}\right., \end{array}$$

The no–slip boundary condition is realized by maintaining the membrane velocity equivalent to that of the surrounding fluid. As the velocity of fluid nodes can be obtained from LBM, the position of the particle membrane node $x_{n}$ can be updated by $u\left(x_{n}\right)=\partial x_{n}/\partial t$. Then, the velocity of membrane nodes $u(x_{n})$ can be calculated according to the local flow velocity $u(x_{f})$

(21) $$u\left(x_{n}\right)=\sum_{f}u\left(x_{f}\right)\delta \left(x_{f}-x_{n}\right),$$

The position of node *n* can be updated according to the motion equation

(22) $$x_{n}\left(t+\Delta t\right)=x_{n}\left(t\right)+u\left(x_{n},t\right)\Delta t.$$

### 2.5. Validation of the Model

**Figure 4:**
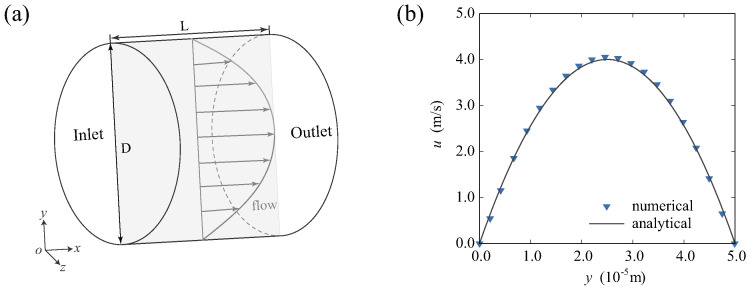
Numerical example of flow field in the cubic channel driven by pressure: (**a**) illustration of the cubic channel; and (**b**) numerical and analytical results of the velocity distribution within the selected cross section.

In order to validate the above model, we chose the Poiseuille flow example in a cylindrical channel shown in Figure 4a. This was performed to verify the flow field and compute the velocity distribution at the radial position of the selected cross section inside the channel. Poiseuille flow is characterized by an irrotational and incompressible flow, with a velocity field that is uniform in all directions. This flow is induced by pressure gradients, exhibiting relative simplicity and symmetry. The flow in the simulation is driven by an external force. The acceleration of the fluid can be obtained by $a=32\nu^{2}Re/D^{3}$, where ν is kinematic viscosity. The length and diameter of the channel are L=D=50μm.

The flow field is segmented into a uniform grid comprising 50 × 50 × 50 units. Parameters used in the simulation can be given as $\Delta x=1.0\times 10^{-6}$ m, $\Delta t=1.5\times 10^{-8}$ s, and $\nu =1.0\times 10^{-6}$ m/s^2^. Re=1000 is considered in this case. Correspondingly, the acceleration of the fluid is $a=2.56\times 10^{4}$ m/s^2^. After completing $4\times 10^{4}$ steps, the radial distribution curve of the flow field is depicted in Figure 4b. The analytical solution for velocity distribution can be presented by

(23) $$u=\frac{\Delta p}{4\mu L}\left(R^{2}-r^{2}\right)=\frac{a}{4\nu }y\left(D-y\right),$$

where *y* is the position in the *y* direction. As illustrated in the figure, it is evident that the results derived using LBM align well with the analytical solution.

**Figure 5:**
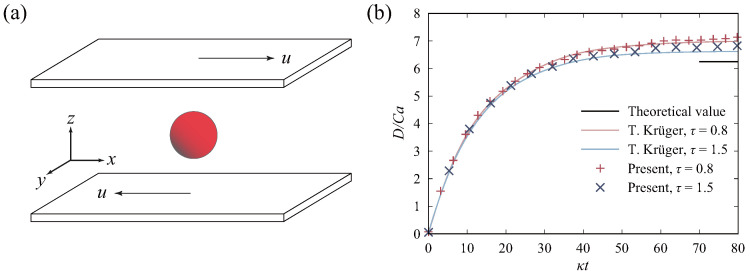
Simulation of particle deformation in shear flow: (**a**) schematic diagram of the particle in shear flow; (**b**) numerical results of particle deformation under varying relaxation time and comparison with those in references.

To verify the accuracy of the interaction between particles and flow in the computational program, we conducted a simulation of particle deformation between two plates. The cubic flow field was divided into a uniform grid of 30 × 30 × 30 units. As illustrated in Figure 5a, the upper and lower plates move at contrasting speeds parallel to the *x* axis. A particle is centrally located within the flow field. It is assumed that the density and viscosity within the particle are the same as those in the flow field. To assess the deformation of particles in the blood flow, we introduced the Taylor shape parameter *D* as an indicator, which can be calculated by

(24) $$D=\frac{A-B}{A+B},$$

where *A* and *B* are the major and minor radii of particles, respectively. The capillary number reflects the ratio of the viscous force to surface tensions of the particle, defined as

(25) $$Ca=\frac{\mu \gamma r_{p}}{\kappa_{s}},$$

where γ is the dimensionless shear rate of the flow, and $r_{p}$ is the initial radius of the particle. The stiffness modulus of the particle in the simulation was set as $\kappa_{s}=4.0\times 10^{-4}$ N/m. The variation of D/Ca with κt and its comparison with the literature values [47] are shown in Figure 5b, where κ=γ/Ca. Upon achieving a steady state in the shear flow, the ratio approaches the theoretical value of 6.25. The figure illustrates that the simulation results of this study align closely with results of references, with the ratio stabilizing around 7. The above results indicate the reliability of the computational program employed in this work.

## 3. Results and Discussion

### 3.1. Migration and Distribution of Particles in the Vessel

In order to study the behavior of individual particles in the blood flow, fewer particles were selected for analysis in the simulation. Initially, two RBCs and two DCs were randomly released in the vessel model. The diameter of the RBC measures 7 μm, while that of DC is 3 μm. The resolution of RBC is 480, and that of DC is 120. The parameters of the RBC and DC model used in this case are presented in Table 2. In the LBM, simulation parameters were assigned as spatial step $\Delta x=1.0\times 10^{-6}$ m, time step $\Delta t=1.8\times 10^{-8}$ s. Correspondingly, the relaxation frequency is 1.78, and Re is 1.465. In Newtonian flow driven by pressure, the particles in the vessel migrate and rotate in accordance with the shear flow. Particles commence movement from random initial positions, migrating towards their equilibrium positions.

**Figure 6:**
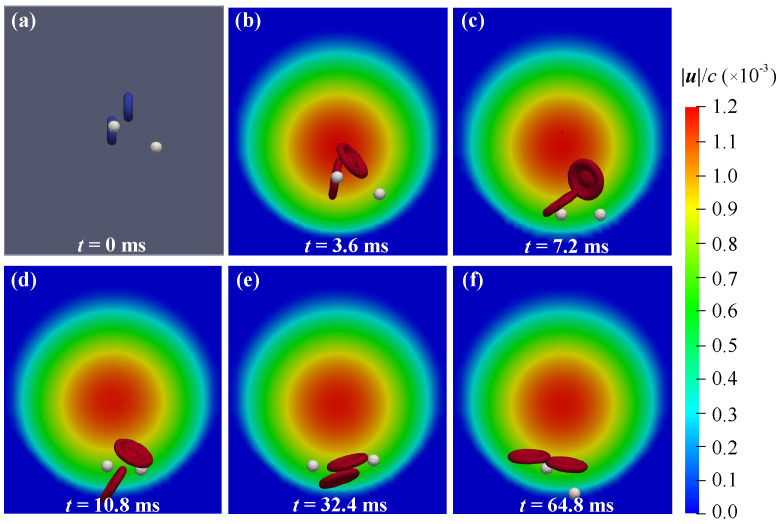
Snapshots of RBCs and DCs in the vessel with the background of velocity distributions on the cross section: (**a**) *t* = 0 ms; (**b**) *t* = 3.6 ms; (**c**) *t* = 7.2 ms; (**d**) *t* = 10.8 ms; (**e**) *t* = 32.4 ms; (**f**) *t* = 64.8 ms.

Figure 6 shows the velocity distribution and particle positions within the cross section of the vessel. We have chosen the flow distribution at the outlet as a reference background with the fluid velocity distribution across the cross section. Particles in the vessel migrate and rotate with the shear flow. The velocity of blood is the highest at the center and gradually decreases towards the vessel wall. The equilibrium regions for both RBCs and DCs are situated in the low–velocity regions near the vessel wall. The velocities of blood flow indicated in the figure are all in lattice units. It can be observed that the maximum flow rate is 0.0012, which corresponds to 0.0667 m/s. In the human circulatory system, the flow velocity of blood in arterioles and venules is approximately several centimeters per second. This also proves that the simulation results in this work align well with the physiological environment. The radial migration of particles is driven by inertial lift force $F_{L}$, which is comprised by a wall–induced inertial lift force $F_{L_{W}}$ and shear–induced inertial lift force $F_{L_{S}}$. The $F_{L_{W}}$ pushes the particle away from the vessel wall, while the $F_{L_{S}}$ pushes the particle away from the vessel center. The particle will attain equilibrium regions with the balance of $F_{L_{W}}$ and $F_{L_{S}}$ [50]. The effect of this balancing is mainly influenced by the settings of flow field and the parameters of DCs.

**Figure 7:**
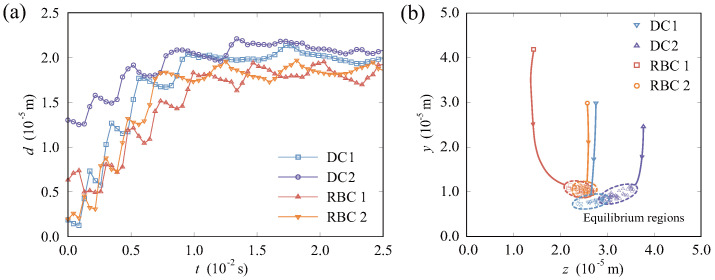
Analysis of RBCs and DCs during migration: (**a**) time histories of the distance *d* for the particles; (**b**) trajectories of particles projected onto the cross section.

In Figure 7, we further studied the motion trajectories of four particles in the system. In the cross section of the vessel illustrated in Figure 7b, we summarized the migration trajectories of particles from their initial positions to equilibrium regions. It shows that smaller DCs exhibit a tendency to concentrate more closer on the vessel wall compared to RBCs. In order to quantify the propensity of particles towards the near–wall regions, as shown in Figure 7a, we defined distance *d* as the linear distance between the center of the vessel and the particle’s center. Following a period of migration and evolution, the values of *d* for RBCs and DCs tend to stabilize around $1.8\times 10^{-5}$ m and $2.0\times 10^{-5}$ m, respectively. In the equilibrium state, minor variations in the *d* value can be attributed to disturbances of the flow field, interactions between particles, random motion, and so on.

### 3.2. Effect of Reynolds Number

In the human circulatory system, both the velocity of blood flow and the Reynolds number exhibit variations across the arteries and veins, as well as within the narrowing and dilating vessels. Meanwhile, blood flow changes with pathological conditions. For instance, hypertension can result in impeded blood flow, leading to a slower flow rate [51]. In this part, we adopted the Reynolds number as a variable to examine their impact on particles’ motion states. We changed Re by regulating the velocity of blood flow. In microvessels, Re is typically low [52]. Accordingly, we set Re to vary from 0.1 to 2.0 in the simulation. Meanwhile, we calculated Re by using the mean velocity in the vessel as reference.

**Figure 8:**
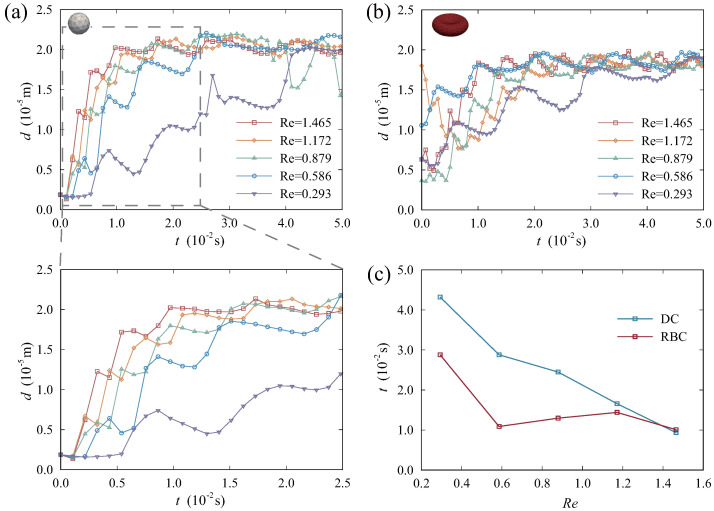
Analysis of the equilibrium regions for RBCs and DCs under varying Reynolds numbers: (**a**) time histories of the distance *d* for DCs; (**b**) time histories of the distance *d* for RBCs; and (**c**) comparison of the time required for RBCs and DCs to reach equilibrium regions.

Figure 8 shows the variations in *d* for DCs and RBCs under different Re, as well as the time required for particles to achieve equilibrium states. After a period of migration, particles are able to attain equilibrium regions and sustain a stable value of *d*. In Figure 8a, it can be observed that the *d* value curve of DCs displays significant fluctuations when Re is relatively low (below 1). This suggests that the migration state of DCs is less stable under these conditions. A more detailed analysis of the transition process from a non–equilibrium state to an equilibrium state can also be observed in Figure 8a. The figure demonstrates that the time required for particles to attain equilibrium regions decreases as Re increases. As shown in Figure 8b, the time for RBCs to migrate to equilibrium regions remains relatively consistent. Nevertheless, the rate of migration significantly decreases when Re=0.293. Concurrently, under conditions of high Re, there is an increased level of randomness in the initial migration stage of RBCs. The curve representing the distance *d* exhibits noticeable fluctuations. In Figure 8c, we contract the time required for DCs and RBCs to achieve equilibrium states. As Re increases, there is a corresponding gradual decrease in the time required for DCs to attain equilibrium regions, thereby exhibiting a more pronounced linear relationship. For RBCs, the time required to reach equilibrium regions is essentially identical under high Re conditions. Under a low Re, the process requires a longer duration. When the Re exceeds approximately 1.2, the time for DCs and RBCs to attain equilibrium regions are very close. As a result, the variation of Re does not significantly influence the equilibrium positions of particles, but it mainly affects the time required for DCs to attain equilibrium states. Under appropriate Re, following their entry into the bloodstream, DCs can attain equilibrium regions proximate to the vessel wall more rapidly. In practical applications, this could promote the rapid arrival of drugs to equilibrium states and facilitate the subsequent specific recognition process of drugs.

**Figure 9:**
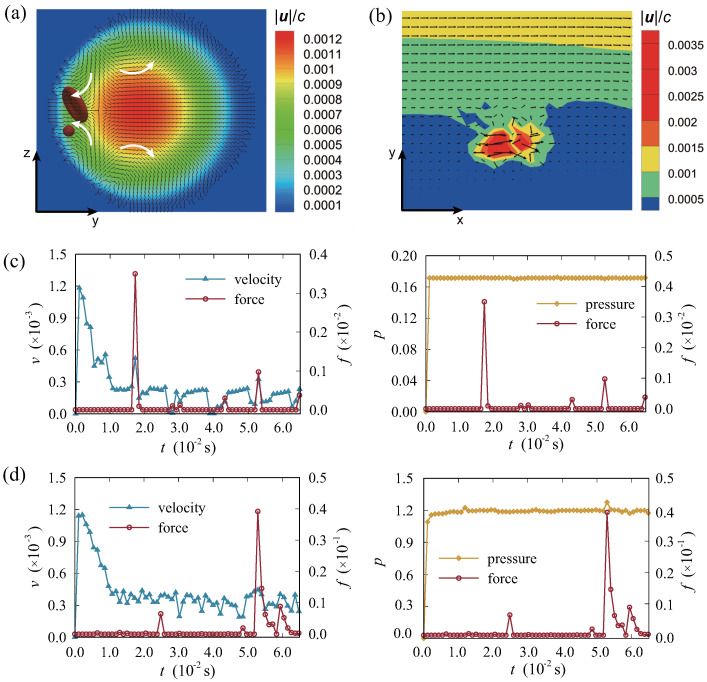
Analysis of the distribution of the fluid field and tangent vectors of flow velocity when Re = 1.465 and *t* = 42.44 ms: (**a**) flow around RBC and DC from the view of the cross section; (**b**) flow around RBC when approaching DC from the view of xoy. Time histories of force, velocity, and pressure of (**c**) DC; (**d**) RBC.

To gain a deeper understanding of the motion states of particles, particles and the fluid field in the cross section are extracted in Figure 9. Here, we studied the detailed interaction between particles and the surrounding flow. Figure 9a shows the flow distribution from the view of cross section. The velocity distribution of the surrounding flow field, where one RBC is subjected to an abrupt force, is illustrated in Figure 9b. It is obvious that the moving RBCs and DCs have an effect on the velocity distribution of the surrounding flows. Figure 9c,d illustrate the alterations in velocity, pressure, and force encountered by RBC and DC throughout their migration process, respectively. Values in this part are all in lattice units. During the motion, there would be varying degrees of abrupt changes in the magnitude of forces. Based on the above results, we speculate that, during the migration, there exists certain interactions between RBCs and DCs. In this study, we employed the magnitude of forces exerted on particle surfaces and the inter–particle distance as comprehensive representations of these effects. These forces in the simulation can be deduced to be interactional, generated between RBCs and DCs. They may manifest as electrostatic forces, hydrophobic interactions, hydrogen bonding and van der Waals forces [53]. Furthermore, a portion of DCs are also able to adhere to the surface of RBC membranes non–specially, which is known as RBC hitchhiking [54,55]. It is worth noting that the absorption process is non–covalent and does not involve any chemical reactions. In our simulation, we aim to investigate the presence of particle interaction during the migration from the mesoscopic perspective.

### 3.3. Effect of Drug Carrier Size

In the development of novel drugs, the sizes of DCs vary significantly owing to different structures and preparation processes. Their sizes typically span the range from nanometers to micrometers. In this part, we focused on the impact of DC size on particle migration and distribution. Firstly, we investigated the temporal validation of the value *d* with different radii of DCs. The radii of DCs range from 1.3 to 2.0 μm (1.3 to 2.0 in lattice unit). Correspondingly, the relaxation frequency is 1.78.

**Figure 10:**
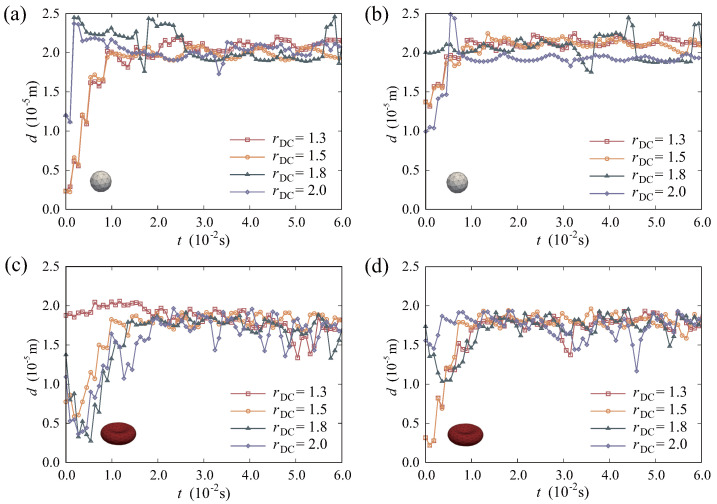
Time histories of the distances *d* for particles with varying sizes: (**a**) DC 1; (**b**) DC 2; (**c**) RBC 1; (**d**) RBC 2.

As shown in Figure 10, all particles eventually migrate to equilibrium regions. As to the stability of particles, both RBCs and DCs are influenced with changes in DC sizes. In Figure 10a,b, it can be observed that the *d* value curve of DCs exhibits a more pronounced fluctuations when the size increases. This suggests that the motion of larger DCs exhibits less stable, while the *d* value curve of RBCs fluctuates greatly in the middle and later period of equilibrium states in Figure 10c,d. Moreover, the extent of instability escalates as the size of DC increases. This further suggests that changes in DC sizes have a significant impact on the migration of RBCs. At the same time, the stability of RBCs is also directly associated with the size of DCs. As for the equilibrium regions of RBCs and DCs, the value *d* of DCs is also influenced by its sizes. In Figure 10a,b, the distance *d* of DCs diminishes as the size of DC expands. This means that the DCs of smaller sizes tend to migrate towards the cell–free layer (CFL) of the vessel. While those of larger sizes stabilize far away from the vessel wall. This not only increases the probability of contact with RBCs, but also promotes their impact on the motion of RBCs. As illustrated in Figure 10c,d, despite fluctuations in the *d* value curve of RBCs, the mean value of *d* shows no significant differences. Overall, the equilibrium regions of RBCs in the system are demonstrated to be stable. But as the size of DCs increases, their equilibrium position approaches the centerline of the vessel. This increases the probability of collision between DCs and RBCs, resulting in larger fluctuations in the movement trajectory of RBCs.

In summary, the variation in DC sizes mainly affect the motion stability of particles in the vessel and the equilibrium positions of DCs. As a result, when it comes to designing DCs for in vivo organismal systems, it is recommended to opt for smaller diameters. Smaller DCs tend to exhibit a more consistent movement and migration patterns within the human body, with relatively minimal interactions and impacts on other cells and substances present in the body.

### 3.4. Effect of Drug Carrier Stiffness

There are plenty of materials suitable for drug carriers, including solid lipid particles, MoF frameworks, polymeric spheres, capsules, micelles, and so on [43]. The stiffness of DCs varies mainly due to their difference in the components. This could also potentially influence their migration process in the bloodstream. Therefore, we investigated the impact of DC stiffness on their motion in this section. The stiffness of particles is regulated by modifying the moduli in Equation (11). Considering the wide range of DC materials, we set the stiffness of DCs larger than those soft RBCs and span eight orders of magnitude. The elastic shear modulus of DCs ranges from $1.0\times 10^{-5}$ to $1.0\times 10^{2}$ N/m. The parameters chosen in the simulation are shown in Table 3.

**Figure 11:**
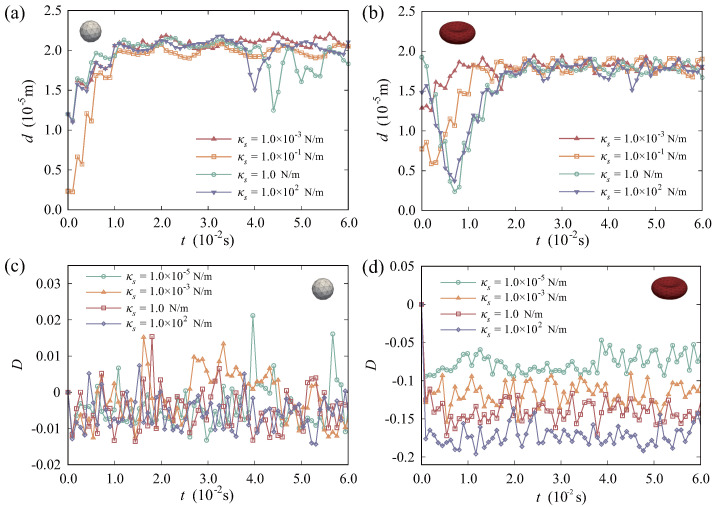
Time histories of the distances *d* for the particles where the stiffness modulus of DCs varies: (**a**) DC; and (**b**) RBC. Time histories of the deformation parameter for particles where the stiffness modulus of DCs varies: (**c**) DC; (**d**) RBC.

Figure 11a,b demonstrate the comparative analysis of the distance *d* of RBCs and DCs over time. Figure 11a illustrates that, after reaching equilibrium stages, DCs remain stable initially, despite changes in DC stiffness. And, their *d* values are very close. However, in the later stage, DCs of higher stiffness reveal instability in the *d* value curve. Considering the average value of *d*, softer DCs tend to migrate towards the vessel wall. Accordingly, as shown in Figure 11b, the migration of RBCs also show similar characteristics. When the stiffness of DCs is relatively higher, there are slightly pronounced fluctuations in the *d* value curves of RBCs. Though the stiffness of DCs varies, the trend of the *d* value curves remains similar after the RBCs reach their equilibrium states. Moreover, with regard to the time required to reach equilibrium states, there appears to be no obvious difference associated with DC stiffness. From this perspective, it can be assumed that alterations in DCs exert minimal impact on the migration stability and equilibrium regions of RBCs within the bloodstream. But, it would influence DCs’ motion stability and trajectories in the later stage of migration.

In order to assess the deformation and interactions of particles in blood flow, we further introduced the Taylor shape parameter with time as a quantitative measure of deformation extent. The deformation parameter D(t) serves to track the deviation of the current shape of a particle from its equilibrium stage. This can be articulated by

(26) $$D\left(t\right)=\frac{\hat{a}\left(t\right)-\hat{b}\left(t\right)}{\hat{a}\left(t\right)+\hat{b}\left(t\right)},\;D\left(t\right)\in \left(-1,1\right),$$

where *a* and *b* are the major and minor radii of the particle, respectively. For deformable particles, the values of *a* and *b* are generally time independent, i.e., a=a(t) and b=b(t). We define $\hat{a}\left(t\right)=a\left(t\right)/a_{0}$ and $\hat{b}\left(t\right)=b\left(t\right)/b_{0}$, where $a_{0}$ and $b_{0}$ represent their undeformed radii. Accordingly, the value of D(t) equals zero when the particle remains undeformed. DCs of three different stiffness moduli had been selected for the analysis of RBC and DC deformation. As shown in Figure 11c, the selected The DCs experience slight deformation during migration, with the absolute value of D(t) less than 2%. Simulation results indicate that softer DCs would exhibit greater deformation compared to the rigid ones. In general, such a small deformation can be ignored in applications. Figure 11d illustrates that, upon reaching equilibrium stages, RBCs also exhibit relatively minimal deformation. However, there are differences among the deformation parameters of the RBCs in several instances. When DCs exhibit higher stiffness, there is a significant increase in the absolute value of the deformation parameter for RBCs, as well. This suggests that DCs with varying stiffness moduli can indeed influence the movement patterns of RBCs in the bloodstream.

To sum up, DCs with varying stiffness obviously affect their motion stability and positions in blood vessels. An increase in DC stiffness may result in a more pronounced deformation of RBCs in their migration process. These simulation results confirm the applicability of DCs made of various components. But when it comes to clinical applications, the design of efficient drug delivery systems necessitates a critical consideration to ensure minimal disruption or damage to normal physiological functions. The results of this section suggest the use of softer DCs to maintain motion stability and reduce the impact on other cells in the system. Additionally, in the future, the precise simulations of diverse DCs would enable researchers to select the carriers of suitable stiffness that align with specific drug attributes and performance requisites.

## 4. Conclusions

In this study, a numerical model has been developed to simulate particle motion within blood vessels through IB–LBM. The aim of this study is to analyze the characteristics of the motion and the distribution of particles in order to encapsule the factors associated with interactions between particles. In our study, we employ Eulerian grids to denote the blood flow field and Lagrangian grids to represent RBCs and DCs within blood vessels. LBM is utilized to address the flow field, while IBM facilitates fluid–solid coupling solutions. Simulations of validation exhibited strong correlation with previous theoretical and numerical studies. We examined the migration and distribution of RBCs and DCs within curved blood vessels and mainly concentrated on the trajectory of individual particles. Firstly, the Reynolds number significantly influences the motion of particles within blood vessels. Compared to RBCs, DCs exhibit a more pronounced response to changes of the Reynolds number. The increase in Re (ranges from 0.2 to 1.2) leads to a shorter time for DCs to reach equilibrium states. This consequently stabilizes the motion of particles. We find that changes in DC size have a vital impact on their movement within the bloodstream. DCs of smaller sizes exhibit a more stable motion state. From the perspective of equilibrium regions, small DCs tend to gravitate towards the vessel wall. Furthermore, the motion stability of DCs directly affects the migration trajectories of RBCs. When DCs in the bloodstream are relatively larger, the motion of RBCs becomes less stable. The variation in membrane stiffness results in a relatively minor difference in the equilibrium positions of particles. In terms of the moving stability of particles, DCs exhibiting greater stiffness may undergo fluctuations during migration, increasing their motion instability. However, the stiffness modulus of DCs does not significantly influence the motion distribution of RBCs. On the basis of stable motion of soft DCs, their interactions with RBCs are weakened, and RBCs become less deformed.

As a result, the properties of blood flow and DCs would significantly influence their equilibrium regions, the time required to reach equilibrium regions, and their motion stability. Consequently, these changes would intensify the interaction between DCs and RBCs, altering the deformation characteristics of RBCs. There is also potential for adsorption to occur between DCs and RBCs. The influence of adsorption among particles within the system warrants further investigation. Additionally, we still need to optimize the model in future work to better simulate the migration of RBCs and DCs in different conditions. In non–Newtonian fluids, an increase in shear rate may result in a decrease in fluid viscosity. This can also lead to complex stress–strain relationships, potentially impacting the trajectories and velocities of RBCs and DCs. Similarly, in pulsatile flows, turbulent phenomena may occur, causing irregular fluid motion. In summary, this work demonstrates the significant application of IB–LBM in the analysis of bioflows within intricate human circulatory systems. Meanwhile, our study illuminates the fundamental mechanisms that dictate DC behavior in intricate geometries, thereby contributing to the advancement of more efficient and precise drug delivery strategies for enhanced therapeutic results.

## Figures and Tables

**Table 1:** Summary of strengths and limitations of several methodologies to investigate blood flow.

Classification	Methodologies	Strengths	Limitations
In vivo experiments	Magnetic resonance imaging (MRI), computed tomography (CT) [16,17,18]	Providing the most real flow field data directly without disturbing the blood flow	Many interference factors; Difficulty in controlling the flow conditions accurately; Lacking reproducibility
In vitro experiments	Dye marking, fluorescence imaging, flow cytometry [19,20]	Ability to carry out systematic quantitative research; High repeatability	Necessity of simplifying model and unable to fully simulate the real physiological environment; High experimental cost
Computational fluid dynamics	Dissipative particle dynamics (DPD), smoothed particle hydrodynamics (SPH), lattice Boltzmann method (LBM)	Enhanced precision and viability in simulating the intricate flow; Strong repeatability and lower cost; Extensive research scale	Necessity of developing reliable models to maintain numerical stability and accuracy

**Table 2:** Parameters of RBCs and DCs used in the simulation.

Parameter	Physical Value	LB Value	References
RBC radius ($r_{R}$)	$3.5\times 10^{-6}$ m	3.5	[46]
RBC elastic shear modulus ($\kappa_{s}$)	$5.0\times 10^{-6}$ N/m	$1.08\times 10^{-6}$	[47,48]
RBC area dilation modulus ($\kappa_{\alpha }$)	$2.5\times 10^{-6}$ N/m	$5.4\times 10^{-6}$	[47,48]
RBC bending modulus ($\kappa_{B}$)	$2.0\times 10^{-19}$ N·m	$4.32\times 10^{-8}$	[47,48]
RBC surface modulus ($\kappa_{A}$)	0.5 N/m	0.108	[47,48]
RBC volume modulus ($\kappa_{V}$)	$1.0\times 10^{6}$ N/m^2^	0.216	[47,48]
DC radius ($r_{D}$)	1.3–2.0 × $10^{-6}$ m	0.8–1.5	—
DC elastic shear modulus ($\kappa_{s}$)	1.0 N/m	0.216	[49]
DC area dilation modulus ($\kappa_{\alpha }$)	0.1 N/m	0.0216	[49]
DC bending modulus ($\kappa_{B}$)	$1.0\times 10^{-13}$ N·m	0.0216	[49]
DC surface modulus ($\kappa_{A}$)	1.0 N/m	0.216	[49]
DC volume modulus ($\kappa_{V}$)	$1.0\times 10^{6}$ N/m^2^	0.216	[49]

**Table 3:** Stiffness moduli for four kinds of DCs.

	$\kappa_{s}$ (N/m)	$\kappa_{\alpha }$ (N/m)	$\kappa_{B}$ (N·m)	$\kappa_{A}$ (N/m)	$\kappa_{V}$ (N/m^2^)
Case 1	$1.0\times 10^{-5}$	$1.0\times 10^{-6}$	$1.0\times 10^{-18}$	$1.0\times 10^{-5}$	$1.0\times 10^{1}$
Case 2	$1.0\times 10^{-3}$	$1.0\times 10^{-4}$	$1.0\times 10^{-16}$	$1.0\times 10^{-3}$	$1.0\times 10^{3}$
Case 3	1.0	$1.0\times 10^{-1}$	$1.0\times 10^{-13}$	1.0	$1.0\times 10^{6}$
Case 4	$1.0\times 10^{1}$	1.0	$1.0\times 10^{-12}$	$1.0\times 10^{1}$	$1.0\times 10^{7}$
Case 5	$1.0\times 10^{2}$	$1.0\times 10^{1}$	$1.0\times 10^{-11}$	$1.0\times 10^{2}$	$1.0\times 10^{8}$

## Data Availability

The data that support the findings of this study are available from the corresponding author upon reasonable request.

## References

[1] Haq Khan Z.U., Khan T.M., Khan A., Shah N.S., Muhammad N., Tahir K., Iqbal J., Rahim A., Khasim S., Ahmad I. (2023). Brief review: Applications of nanocomposite in electrochemical sensor and drugs delivery. Front. Chem..

[2] Mitchell M.J., Billingsley M.M., Haley R.M., Wechsler M.E., Peppas N.A., Langer R. (2021). Engineering precision nanoparticles for drug delivery. Nat. Rev. Drug Discov..

[3] Sharma M., Dev S.K., Kumar M., Shukla A.K. (2018). Microspheres as suitable drug carrier in sustained release drug delivery: An overview. Asian J. Pharm. Pharmacol..

[4] Medina-Sánchez M., Xu H., Schmidt O.G. (2018). Micro-and nano-motors: The new generation of drug carriers. Ther. Deliv..

[5] Avsievich T., Popov A., Bykov A., Meglinski I. (2019). Mutual interaction of red blood cells influenced by nanoparticles. Sci. Rep..

[6] Sun Y., Su J., Liu G., Chen J., Zhang X., Zhang R., Jiang M., Qiu M. (2017). Advances of blood cell-based drug delivery systems. Eur. J. Pharm. Sci..

[7] Lenders V., Escudero R., Koutsoumpou X., Armengol Álvarez L., Rozenski J., Soenen S.J., Zhao Z., Mitragotri S., Baatsen P., Allegaert K. (2022). Modularity of RBC hitchhiking with polymeric nanoparticles: Testing the limits of non-covalent adsorption. J. Nanobiotechnol..

[8] Muzykantov V.R. (2010). Drug delivery by red blood cells: Vascular carriers designed by mother nature. Expert Opin. Drug Deliv..

[9] Forouzandehmehr M., Ghoytasi I., Shamloo A., Ghosi S. (2022). Particles in coronary circulation: A review on modelling for drug carrier design. Mater. Des..

[10] Sahai N., Gogoi M., Ahmad N. (2021). Mathematical modeling and simulations for developing nanoparticle-based cancer drug delivery systems: A review. Curr. Pathobiol. Rep..

[11] Mircioiu C., Voicu V., Anuta V., Tudose A., Celia C., Paolino D., Fresta M., Sandulovici R., Mircioiu I. (2019). Mathematical modeling of release kinetics from supramolecular drug delivery systems. Pharmaceutics.

[12] Zeng L., An L., Wu X. (2011). Modeling drug-carrier interaction in the drug release from nanocarriers. J. Drug Deliv..

[13] Hamilton S., Kingston B.R. (2024). Applying artificial intelligence and computational modeling to nanomedicine. Curr. Opin. Biotechnol..

[14] Pushkaran A.C., Arabi A.A. (2024). From understanding diseases to drug design: Can artificial intelligence bridge the gap?. Artif. Intell. Rev..

[15] Kibria M.R., Akbar R.I., Nidadavolu P., Havryliuk O., Lafond S., Azimi S. (2023). Predicting efficacy of drug-carrier nanoparticle designs for cancer treatment: A machine learning-based solution. Sci. Rep..

[16] Pavel D.G., Zimmer A.M., Patterson V.N. (1977). In vivo labeling of red blood cells with 99mTc: A new approach to blood pool visualization. J. Nucl. Med..

[17] Shah R., Bag A., Chapman P., Curé J. (2010). Imaging manifestations of progressive multifocal leukoencephalopathy. Clin. Radiol..

[18] Tarakanchikova Y., Stelmashchuk O., Seryogina E., Piavchenko G., Zherebtsov E., Dunaev A., Popov A., Meglinski I. (2018). Allocation of rhodamine-loaded nanocapsules from blood circulatory system to adjacent tissues assessed in vivo by fluorescence spectroscopy. Laser Phys. Lett..

[19] Pérez-López A., Torres-Suárez A.I., Martín-Sabroso C., Aparicio-Blanco J. (2023). An overview of in vitro 3D models of the blood–brain barrier as a tool to predict the in vivo permeability of nanomedicines. Adv. Drug Deliv. Rev..

[20] Koonyosying P., Srichairatanakool S., Tiwananthagorn S., Sthitmatee N. (2023). Measurement of Babesia bovis infected red blood cells using flow cytometry. J. Microbiol. Methods.

[21] Ye T., Phan-Thien N., Lim C.T., Peng L., Shi H. (2017). Hybrid smoothed dissipative particle dynamics and immersed boundary method for simulation of red blood cells in flows. Phys. Rev. E.

[22] Dzwinel W., Boryczko K., Yuen D.A. (2003). A discrete-particle model of blood dynamics in capillary vessels. J. Colloid Interface Sci..

[23] Soleimani M., Sahraee S., Wriggers P. (2019). Red blood cell simulation using a coupled shell–fluid analysis purely based on the SPH method. Biomech. Model. Mechanobiol..

[24] Polwaththe-Gallage H.N., Saha S.C., Sauret E., Flower R., Gu Y. (2016). A coupled SPH-DEM approach to model the interactions between multiple red blood cells in motion in capillaries. Int. J. Mech. Mater. Des..

[25] Wang S., Ma S., Li R., Qi X., Han K., Guo L., Li X. (2023). Probing the interaction between supercarrier rbc membrane and nanoparticles for optimal drug delivery. J. Mol. Biol..

[26] Djukic T., Filipovic N.D. (2021). Modeling the Motion of Rigid and Deformable Objects in Fluid Flow. Computational Modeling and Simulation Examples in Bioengineering.

[27] Peskin C.S. (1972). Flow patterns around heart valves: A numerical method. J. Comput. Phys..

[28] Sun C., Munn L.L. (2005). Particulate nature of blood determines macroscopic rheology: A 2-D lattice Boltzmann analysis. Biophys. J..

[29] Sun C., Munn L.L. (2008). Lattice-Boltzmann simulation of blood flow in digitized vessel networks. Comput. Math. Appl..

[30] Skorczewski T., Erickson L.C., Fogelson A.L. (2013). Platelet motion near a vessel wall or thrombus surface in two-dimensional whole blood simulations. Biophys. J..

[31] Kaoui B. (2018). Computer simulations of drug release from a liposome into the bloodstream. Eur. Phys. J. E.

[32] Ye H., Shen Z., Li Y. (2017). Cell stiffness governs its adhesion dynamics on substrate under shear flow. IEEE Trans. Nanotechnol..

[33] Ye H., Shen Z., Li Y. (2018). Shear rate dependent margination of sphere-like, oblate-like and prolate-like micro-particles within blood flow. Soft Matter.

[34] Ye H., Shen Z., Li Y. (2019). Shape-dependent transport of microparticles in blood flow: From margination to adhesion. J. Eng. Mech..

[35] Ye H., Shen Z., Li Y. (2019). Interplay of deformability and adhesion on localization of elastic micro-particles in blood flow. J. Fluid Mech..

[36] Ye H., Shen Z., Wei M., Li Y. (2021). Red blood cell hitchhiking enhances the accumulation of nano-and micro-particles in the constriction of a stenosed microvessel. Soft Matter.

[37] Yue K., You Y., Yang C., Niu Y., Zhang X. (2020). Numerical simulation of transport and adhesion of thermogenic nano-carriers in microvessels. Soft Matter.

[38] Nikfar M., Razizadeh M., Paul R., Muzykantov V., Liu Y. (2021). A numerical study on drug delivery via multiscale synergy of cellular hitchhiking onto red blood cells. Nanoscale.

[39] Venier-Julienne M., Benoit J. (1996). Preparation, purification and morphology of polymeric nanoparticles as drug carriers. Pharm. Acta Helv..

[40] Scioli Montoto S., Muraca G., Ruiz M.E. (2020). Solid lipid nanoparticles for drug delivery: Pharmacological and biopharmaceutical aspects. Front. Mol. Biosci..

[41] Trucillo P. (2021). Drug carriers: Classification, administration, release profiles, and industrial approach. Processes.

[42] Kavita K., Ashvini V., Ganesh N. (2010). Albumin microspheres. Unique system as drug delivery carriers for non steroidal anti inflammatory drugs. Int. J. Pharm. Sci. Rev. Res..

[43] García M.C. (2020). Nano-and microparticles as drug carriers. Engineering Drug Delivery Systems.

[44] Krüger T., Varnik F., Raabe D. (2011). Efficient and accurate simulations of deformable particles immersed in a fluid using a combined immersed boundary lattice Boltzmann finite element method. Comput. Math. Appl..

[45] Ramanujan S., Pozrikidis C. (1998). Deformation of liquid capsules enclosed by elastic membranes in simple shear flow: Large deformations and the effect of fluid viscosities. J. Fluid Mech..

[46] Skalak R., Tozeren A., Zarda R., Chien S. (1973). Strain Energy Function of Red Blood Cell Membranes. Biophys. J..

[47] Krüger H. (2012). Computer Simulation Study of Collective Phenomena in Dense Suspensions of Red Blood Cells Under Shear.

[48] Gompper G., Schick M. (2008). Soft Matter: Lipid Bilayers and Red Blood Cells.

[49] Sun D.K., Wang Y., Dong A.P., Sun B.D. (2016). A three-dimensional quantitative study on the hydrodynamic focusing of particles with the immersed boundary–Lattice Boltzmann method. Int. J. Heat Mass Transf..

[50] Jiang D., Ni C., Tang W., Xiang N. (2020). Numerical simulation of elasto-inertial focusing of particles in straight microchannels. J. Phys. D Appl. Phys..

[51] Wiggers C.J. (1938). The dynamics of hypertension. Am. Heart J..

[52] Secomb T.W. (2017). Blood flow in the microcirculation. Annu. Rev. Fluid Mech..

[53] Nguyen P.H.D., Jayasinghe M.K., Le A.H., Peng B., Le M.T.N. (2023). Advances in Drug Delivery Systems Based on Red Blood Cells and Their Membrane-Derived Nanoparticles. ACS Nano.

[54] Chambers E., Mitragotri S. (2004). Prolonged circulation of large polymeric nanoparticles by non-covalent adsorption on erythrocytes. J. Control Release.

[55] Chambers E., Mitragotri S. (2007). Long Circulating Nanoparticles via Adhesion on Red Blood Cells: Mechanism and Extended Circulation. Exp. Biol. Med..

